# Perinatal outcomes and uptake of RSV vaccine during pregnancy in South London: a cross-sectional study

**DOI:** 10.1136/bmjopen-2025-101592

**Published:** 2025-09-24

**Authors:** Mohammad Sharif Razai, Erkan Kalafat, Smriti Prasad, Chelone Lee-Wo, Paul T Heath, Asma Khalil

**Affiliations:** 1 Primary Care Unit, Department of Public Health and Primary Care, University of Cambridge, Cambridge, UK; 2 Division of Reproductive Endocrinology and Infertility, Department of Obstetrics and Gynecology, Koç Üniversitesi, Istanbul, Türkiye; 3 Vascular Biology Research Centre, Molecular and Clinical Sciences Research Institute, City St George’s, University of London, London, England, UK; 4 Department of Obstetrics and Gynaecology, St George’s NHS Trust, London, UK; 5 Vaccine Institute, Institute of Infection and Immunity, City St Georges, University of London, London, UK; 6 Royal College of Obstetricians and Gynaecologists, London, UK

**Keywords:** Public health, Immunology, Infection control

## Abstract

**Abstract:**

**Background:**

Maternal respiratory syncytial virus (RSV) vaccination has been introduced to protect infants from severe respiratory infections. However, its uptake and impact on perinatal outcomes are unknown in the UK.

**Objectives:**

To evaluate uptake of RSV vaccine during pregnancy in a UK population.

**Methods and analysis:**

This cross-sectional study was conducted at a tertiary maternity hospital in London. The participants included pregnant women who delivered between 1 September and 17 December 2024 (n=1157). For the analysis of vaccine uptake, the cohort included women eligible for vaccination who delivered beyond 28 weeks’ gestation and were at 36 weeks or less on 1 September 2024 (n=911). The main outcome measures were RSV vaccine uptake and its association with sociodemographic factors, perinatal outcomes including preterm birth (PTB), hypertensive disorders of pregnancy and stillbirth.

**Results:**

Of 911 eligible women, 19% (n=173) received the RSV vaccine during pregnancy. Uptake increased significantly from 4% in September to 32% in December (p<0.001). Vaccinated women were older (median age 35 vs 32 years, p<0.001), more likely to be white (66.3% vs 46.5%, p<0.001), and from higher socioeconomic quintiles (Index of Multiple Deprivation quintile 5: 18.5% vs 11.2%, p=0.029). Smoking during pregnancy was less common in vaccinated women (0.6% vs 5.0%, p=0.016). Multivariable logistic regression identified older maternal age (p=0.001), higher socioeconomic status (IMD quintile 1 vs 5; adjusted OR (aOR) 0.28, 95% CI 0.08 to 0.81, p=0.030) and ethnicity (Asian: aOR 0.56, 95% CI 0.35 to 0.88, p=0.015, black: aOR 0.52, 95% CI 0.28 to 0.95, p=0.040, mixed: aOR 0.12, 95% CI 0.02 to 0.39, p=0.004) as independent predictors of vaccine uptake. PTB rates at <37 weeks were comparable between groups (9.6% vs 10.0%, p=0.980), but no vaccinated women experienced PTB<34 weeks compared with 3.2% of unvaccinated women (p=0.032). Caesarean section rates were higher among vaccinated women (52.0% vs 35.5%, p<0.001). No significant differences were observed in other perinatal outcomes.

**Conclusions:**

RSV vaccine uptake shows significant increases over time, with disparities in uptake by ethnicity and socioeconomic status. Further research is needed to increase vaccination rates, particularly in disadvantaged groups, and evaluate perinatal outcomes.

**STRENGTHS AND LIMITATIONS OF THIS STUDY**

- First UK study to report the association between respiratory syncytial virus vaccine uptake in pregnancy and sociodemographic and perinatal outcomes.
- Large cohort enabled comparison of vaccinated and unvaccinated women with detailed clinical and demographic data.
- Cross-sectional, single-centre design may limit generalisability and introduces risk of immortal time bias.
- Perinatal outcomes were based on unadjusted comparisons, with potential for residual confounding.

## Introduction

 Respiratory syncytial virus (RSV) is a major cause of infant morbidity and mortality. In the UK, it accounts for approximately 33 500 hospitalisations in children under 5 years old and 20 to 30 deaths annually.^1^ Across the EU, Norway and the UK, it causes an estimated 213 000 hospitalisations in children under 5 years old.^2^ RSV infects up to 90% of children within their first 2 years of life and frequently reinfects older children.^1^

The RSV vaccine is expected to prevent up to 5000 hospitalisations and 15 000 emergency department visits annually in the UK, reducing the risk of severe bronchiolitis by 70% during the first 6 months of life.^3 4^ However, concerns about a higher rate of preterm birth (PTB) associated with RSV vaccines were raised during phase 3 clinical trials, with a non-significant 1% increase observed in the (RSVpreF) trial^5^ and a 1.9% increase in another trial (RSVPreF3-Mat), which led to it being halted.^6^ The EU approved RSV vaccination for use in pregnancy in 2023; however, its implementation varies across the region.^2^ Abrysvo (RSVpreF), the bivalent RSV vaccine developed by Pfizer, is the only maternal vaccine licensed in the UK by the Medicines and Healthcare products Regulatory Agency since November 2023 following phase 3 clinical trials. From September 2024, the UK Joint Committee on Vaccination and Immunisation (JCVI) recommends receiving one dose of RSV vaccine in every pregnancy from 28 weeks’ gestation,^3^ while in France, for example, it is recommended between 32 and 36 weeks’ gestation.^7^ This variation reflects differing views on the immunogenicity and safety of the vaccine. In the NHS, the RSV maternal vaccination programme was introduced as part of routine antenatal care, delivered primarily through hospital-based maternity units, with additional access at selected primary care sites. RSV vaccination in pregnancy is not seasonal and can be offered year-round to individuals who are at least 28 weeks pregnant. Although the vaccine can be given up to the point of delivery, immunisation after 36 weeks may not provide the same level of passive protection to the infant, as there may be insufficient time for the mother to mount an effective immune response and transfer antibodies across the placenta.^8^

The JCVI also advises RSV monoclonal antibody immunisation, nirsevimab or palivizumab, for high-risk infants. Nirsevimab will be available through a national immunisation programme in the UK for the 2025 RSV season. High-risk groups include preterm infants with chronic lung disease; infants with ongoing oxygen needs due to conditions like pulmonary hypoplasia, congenital lung anomalies and interstitial lung disease; those with haemodynamically significant congenital heart disease and children under 24 months with severe combined immunodeficiency.^9^ Moreover, long-acting monoclonal antibodies will be offered to very preterm infants (born <32 weeks) ahead of their first RSV season (when available), as they may not benefit from maternal vaccination.^9^

The maternal RSV vaccination rate of 33.6% and 53.1% was reported for September 2024 and January 2025, respectively, in England.^10 11^ This study was conducted due to limited data on RSV vaccination coverage and safety since its introduction.

## Material and methods

We conducted a cross-sectional study of women who gave birth at a tertiary maternity hospital in London between 1 September and 17 December 2024 (n=1157). The hospital serves a diverse ethnic and socioeconomic population of approximately 1.3 million people in South West London. This period was selected to capture variations in uptake following the vaccine’s introduction and to align with the RSV season, which begins in October and peaks in December.^12^ In tertiary settings, midwives usually offer RSV vaccination to pregnant women in antenatal clinics, including during routine scans, via drop-in clinics and opportunistically during routine or urgent hospital admissions. In the community, some primary care sites and midwife-led clinics also provide RSV vaccination.

Data also included perinatal outcomes between those who received the RSV vaccine in pregnancy and those who did not. Perinatal outcomes included PTB (<37, <34 and <32 weeks), hypertensive disorders of pregnancy (HDP), mode of delivery, intrapartum chorioamnionitis, intrapartum fever, placental abruption, postpartum haemorrhage, maternal admission to a high dependency unit (HDU), fetal abnormalities,^13^ small-for-gestational age newborn, stillbirth (fetal death ≥24 weeks’ gestation) and neonatal unit (NNU) admission. HDP were identified through clinician-recorded diagnoses in the electronic medical record according to the 2021 International Society for the Study of Hypertension in Pregnancy guidelines.^14^ To ensure accuracy, a chart review was conducted to confirm that the documented diagnoses met the criteria outlined in the guidelines. In this cross-sectional analysis, all variables, including HDP, were ascertained for the entire pregnancy following delivery. These variables are presented to describe differences between vaccinated and unvaccinated women and, where relevant, to explore associations with vaccine uptake. No temporal or causal relationship with vaccination was assessed for HDP.

Small for gestational age was classified as a birth weight below the 10th percentile using UK-WHO growth charts. Intrapartum chorioamnionitis was identified based on a clinician-assigned diagnosis coded in the electronic medical records. A nurse or midwife abstracted data. Diagnosis was made by the attending clinician based on clinical observations and a combination of factors, including maternal tachycardia, a maternal temperature of ≥38.0°C on a single reading or ≥37.5°C on two consecutive occasions at least 1 hour apart, raised inflammatory markers and fetal tachycardia.^15^ Postpartum haemorrhage was defined as the loss of 500 mL or more of blood from the genital tract following childbirth.^16^ Placental abruption was diagnosed clinically in accordance with the Royal College of Obstetricians and Gynaecologists guidance on the management of antepartum haemorrhage.^17^ The fetal abnormality outcome included conditions screened for under the NHS Fetal Anomaly Screening Programme, such as structural anomalies (eg, open spina bifida, congenital heart defects) and chromosomal abnormalities (eg, trisomy 21, 18 and 13).^13 18^

When estimating the uptake of the RSV vaccine, the eligibility criteria included all pregnancies delivering beyond 28 weeks’ gestation and who were at 36 weeks or less on 1 September 2024 (n=911). Medical records contained data on sociodemographic variables obtained at the first hospital-based antenatal (booking) visit during pregnancy (eg, age, ethnicity and socioeconomic status), clinical factors (eg, body mass index (BMI), obesity, parity, smoking status and alcohol consumption) and perinatal outcomes. Uptake of the bivalent RSV prefusion F (RSVpreF) protein-based (Pfizer) vaccine was defined as documented receipt during pregnancy. The IMD quintiles are used in the UK to classify areas based on socioeconomic deprivation. It ranks neighbourhoods from quintile 1 (most deprived) to quintile 5 (least deprived), considering factors such as income, education, health and housing. Ethnicity is self-reported at the time of registration.

As this research involved a secondary analysis of anonymised data from routine clinical care records for service evaluation, it did not require ethical review.

Descriptive statistics were calculated for vaccinated and unvaccinated groups, and differences were assessed using the χ^2^ test for categorical variables and the Mann-Whitney U test for continuous variables. Logistic regression was performed to identify independent predictors of vaccine uptake, adjusting for relevant covariates. The results were reported as adjusted ORs (aORs) and their 95% CIs. Missingness was addressed with multivariate imputation with chained equations. Statistical significance was set at p<0.05. We followed the STrengthening the Reporting of OBservational studies in Epidemiology (STROBE) reporting guidelines.

## Results

Of 911 women eligible for vaccination, 19% (173) received the RSV vaccine during pregnancy between 1 September and 17 December 2024 (table 1). Vaccine uptake increased significantly each month: 4% in September (5/121), 17% in October (54/322), 20% in November (58/293) and 32% in December (56/175) (Cochran-Armitage test for trend, p<0.001). Vaccinated women were significantly older (p<0.001, table 1). Women from higher socioeconomic quintiles (IMD 5) were more likely to be vaccinated (18.5% vs 11.2%). Ethnicity was also significantly associated with vaccine uptake: 66.3% of vaccinated women were white, compared with 46.5% in the unvaccinated group. Uptake was lower among Asian women (18.1%, p<0.001) and black women (9.6%). Only 0.6% of vaccinated women were smokers compared with 5.0% in the unvaccinated group (p=0.016). No significant differences were observed for BMI, obesity, multiple pregnancies, alcohol consumption, HDP or diabetes between vaccinated and unvaccinated women (all p>0.05, table 1). Median gestational age at delivery was similar between RSV and non-RSV-vaccinated women: 39.15 weeks (IQR 38.00–40.00) vs 39.12 weeks (IQR 38.00–40.00), respectively (p=0.056).

**Table 1:** Sociodemographic and clinical characteristics of 911 eligible pregnant women by respiratory syncytial virus (RSV) vaccine uptake status

Variables	Unvaccinated (n=738)	Vaccinated (n=173)	P value
Maternal age in years, median (IQR)	32.0 (29.0 to 36.0)	35.0 (32.0 to 37.0)	<0.001
Multiparous, n (%)	382 (51.8)	85 (49.1)	0.591
Index of Multiple Deprivation (IMD), median (IQR)	6.0 (4.0 to 7.0)	6.0 (4.0 to 8.0)	0.011
IMD quintile, n (%)			0.029
5 (least deprived)	83 (11.2)	32 (18.5)	
4	169 (22.9)	39 (22.5)	
3	214 (29.0)	53 (30.6)	
2	220 (29.8)	44 (25.4)	
1 (most deprived)	52 (7.0)	5 (2.9)	
Race and ethnicity, n (%)			<0.001
White	322 (46.5)	110 (66.3)	
Asian	175 (25.3)	30 (18.1)	
Black	100 (14.4)	16 (9.6)	
Mixed	51 (7.4)	2 (1.2)	
Others	45 (6.5)	8 (4.8)	
Maternal body mass index in Kg/m^2^, median (IQR)	25.0 (22.0 to 29.0)	26.0 (22.0 to 29.0)	0.894
Obese, n (%)	170 (23.0)	36 (20.8)	0.597
Smoker during pregnancy, n (%)	37 (5.0)	1 (0.6)	0.016
Alcohol consumption in pregnancy, n (%)	5 (0.7)	1 (0.6)	>0.999
Pre-pregnancy diabetes, n (%)	5 (0.7)	1 (0.6)	>0.999
Hypertensive disorders in pregnancy, n (%)	45 (6.1)	13 (7.5)	0.607
Multiple pregnancy, n (%)	30 (4.1)	8 (4.6)	0.905

IQR, Interquartile Range.

Multivariable logistic regression (table 2) identified older maternal age (p=0.001), higher socioeconomic status (IMD quintile 1 vs 5; aOR 0.28, 95% CI 0.08 to 0.81, p=0.030) and ethnicity (Asian: aOR 0.56, 95% CI 0.35 to 0.88, p=0.015, black: aOR 0.52, 95% CI 0.28 to 0.95, p=0.040, Mixed: aOR 0.12, 95% 0.02 to 0.39, p=0.004) as independent predictors of vaccine uptake. White ethnicity was the reference group. Smoking remained a significant negative predictor (p=0.043).

**Table 2:** Multivariable analysis of factors associated with RSV vaccine uptake in pregnancy

Variable	Unadjusted OR (95% CI, P value)	Adjusted OR (95% CI, P value)
Maternal age in years	**1.08 (1.05–1.12, p<0.001)**	**1.07 (1.03–1.11, p=0.001)**
Multiparity	0.90 (0.65–1.25, p=0.534)	0.89 (0.62–1.27, p=0.523)
IMD quintile		
5 (least deprived)	Reference	Reference
4	0.60 (0.35–1.03, p=0.061)	0.64 (0.36–1.13, p=0.125)
3	0.64 (0.39–1.07, p=0.087)	0.64 (0.37–1.10, p=0.103)
2	**0.52 (0.31–0.88, p=0.013)**	0.68 (0.39–1.19, p=0.172)
1 (most deprived)	**0.25 (0.08–0.63, p=0.007)**	**0.28 (0.08–0.81, p=0.030)**
Race and ethnicity		
White	Reference	Reference
Asian	**0.50 (0.32–0.77, p=0.002)**	**0.56 (0.35–0.88, p=0.015)**
Black	**0.47 (0.26–0.81, p=0.009)**	**0.52 (0.28–0.95, p=0.040)**
Mixed	**0.11 (0.02–0.38, p=0.003)**	**0.12 (0.02–0.39, p=0.004)**
Others	0.52 (0.22–1.08, p=0.102)	0.54 (0.23–1.15, p=0.132)
Body mass index	0.99 (0.96–1.02, p=0.633)	1.01 (0.96–1.06, p=0.662)
Obesity	0.88 (0.58–1.30, p=0.529)	0.93 (0.48–1.82, p=0.764)
Smoker in pregnancy	**0.11 (0.01–0.51, p=0.030)**	**0.12 (0.01–0.60, p=0.043)**
Alcohol consumption in pregnancy	0.85 (0.04–5.33, p=0.884)	1.45 (0.07–10.29, p=0.744)
Diabetes mellitus	0.85 (0.04–5.33, p=0.884)	1.90 (0.09–17.39, p=0.599)
Hypertension in pregnancy	1.25 (0.63–2.31, p=0.493)	1.29 (0.63–2.53, p=0.469)
Multiple pregnancy	1.14 (0.48–2.43, p=0.741)	1.11 (0.45–2.50, p=0.808)

Effect estimates with significant P values are typed in bold.

CI, Confidence Interval ; IMD, Index of Multiple Deprivation; OR, Odds Ratio; RSV, respiratory syncytial virus.

The rate of PTB<37 weeks was comparable between vaccinated and unvaccinated women (10.0% vs 9.6%, p=0.980). No PTB <34 weeks or <32 weeks occurred in the vaccinated group, compared with 3.2% and 2.3% in the unvaccinated group, respectively, though these differences were not statistically significant at <32 weeks (p=0.077) but significant at <34 weeks (p=0.032). There was no significant difference in the incidence of HDP between vaccinated and unvaccinated women (7.5% vs 6.1%, p=0.607). Caesarean section (CS) was more common among vaccinated women (52.0% vs 35.5% in unvaccinated women, p<0.001), while unassisted vaginal deliveries were less frequent in vaccinated women (36.2% vs 51.1%) in unvaccinated women. The rate of intrapartum chorioamnionitis was significantly lower in the vaccinated group (0.6% in vaccinated vs 4.0% in unvaccinated, p=0.039). No significant differences were observed in other maternal and neonatal outcomes, including intrapartum fever (2.8% in vaccinated vs 4.4% in unvaccinated, p=0.450), placental abruption (1.1% in vaccinated vs 0.5% in unvaccinated, p=0.651), postpartum haemorrhage (11.9% in vaccinated vs 12.9% in unvaccinated, p=0.808) or maternal admission to a HDU (3.4% in vaccinated vs 5.1% in unvaccinated, p=0.432). There was no significant difference in fetal abnormalities (2.3% in vaccinated vs 1.9% in unvaccinated, p=0.999), small-for-gestational-age newborn (10.7% in vaccinated vs 13.3% in unvaccinated, p=0.422), stillbirth or neonatal death (none in vaccinated vs 1.1% in unvaccinated, p=0.320) or NNU admission (6.2% in vaccinated vs 6.3% in unvaccinated, p>0.999).

## Discussion

Older maternal age, higher socioeconomic status and white ethnicity were associated with higher vaccine uptake, while smoking and minority ethnic status were associated with lower coverage. There were no significant differences in perinatal outcomes between vaccinated and unvaccinated women, including HDP, admission to HDU or NNU, stillbirth, neonatal death or PTB. In fact, unvaccinated women had higher rates of PTB <34 weeks compared with vaccinated women.

The RSV vaccine uptake in December (32%) aligns with data from the USA, where vaccine coverage among pregnant women during the 2023–2024 season was 33%. Low vaccine uptake has similarly been observed for other maternal vaccines, including influenza, pertussis and COVID-19.^19 20^ Our findings of lower uptake among ethnic minority women and those from socioeconomically deprived backgrounds are consistent with previous research on maternal vaccinations.^19 20^ Uptake increased over time as vaccine delivery systems became more established. Early in the rollout, limited staff awareness, a lack of training in vaccine preparation and changing clinic workflows likely contributed to lower uptake.

In contrast to concerns raised in RSV vaccine clinical trials regarding a higher risk of PTB,^5 6^ our study found no increased risk of PTB among vaccinated women. Additionally, our study did not find increased risk of HDP, whereas a prior study using time-dependent models reported an elevated risk (HR, 1.43; 95% CI 1.16 to 1.77).^21^ However, our study was not prospectively designed or powered to assess vaccine safety; therefore, any observed associations should be interpreted with particular caution.

As of August 2025, over 337,000 pregnant women in England had received the RSV vaccine.^22^ Notably, vaccination rates have been increasing over time; however, uptake remains suboptimal, particularly among ethnic minority women.^11 23^ In January 2025, the highest coverage was reported among Chinese (75%), followed by White Irish and British groups (63% and 61%, respectively). The lowest coverage was observed in Black Caribbean (28.2%), Black or Black British (32.5%) and Asian or Asian British–Pakistani (33.4%) groups.^11^ Additionally, London had the lowest RSV vaccine coverage (44%) while South East England had the highest (62.7%).^11^ Further work is required to achieve the coverage necessary to have a maximal impact on UK infant disease. The higher CS rate in vaccinated women may reflect their older age and higher socioeconomic status, factors associated with increased elective or clinically indicated CS.

This study provides the first UK data, to our knowledge, on RSV vaccine uptake and its association with sociodemographic factors and perinatal outcomes in pregnancy. The large cohort size allows for robust comparisons between vaccinated and unvaccinated women. Monthly trend data highlight patterns in vaccine uptake over time. However, limitations include the use of a single centre, which may limit generalisability to other populations. The cross-sectional design and inclusion of only women who delivered during a defined period may have introduced immortal time bias. Specifically, women at later gestational ages on 1 September 2024 had less opportunity to receive the RSV vaccine before delivery, potentially underestimating uptake and biasing associations with perinatal outcomes. Immortal time bias may also explain the absence of PTBs <34 weeks in the vaccinated group. Given this, comparisons of perinatal outcomes, particularly those sensitive to gestational age, should be interpreted with caution, as this bias may have influenced the observed differences between groups. The comparison of perinatal outcomes between vaccinated and unvaccinated groups was based on crude rates and unadjusted analyses. Without time-adjusted or multivariable models to account for differences in gestational age at vaccination and delivery, residual confounding may affect the interpretation of vaccine safety outcomes. Furthermore, although some differences in outcomes (eg, PTB at <34 weeks) were observed, the study was not prospectively designed or powered to assess the safety of the RSV vaccine. Finally, data on neonatal RSV-related morbidity, such as emergency department visits or hospitalisations, were not available, which restricts evaluation of the vaccine’s effectiveness in preventing neonatal disease.

## Conclusions

RSV vaccine uptake in pregnancy is increasing over time, with disparities by ethnicity and socioeconomic status. Findings suggest that vaccination was not associated with adverse perinatal outcomes. Further research is needed to explore interventions to improve vaccine coverage, particularly among socioeconomically and ethnically disadvantaged groups, and to evaluate perinatal outcomes.

## Data Availability

Data are available upon reasonable request.
