# Supplementary material for: Perinatal outcomes and uptake of RSV vaccine during pregnancy in South London: a cross-sectional study
Source: BMJ Open. 2025 Sep 24;15(9):e101592. doi: 10.1136/bmjopen-2025-101592 (PMC12481338; doi:10.1136/bmjopen-2025-101592)
Supplement: Supplementary file 1 [file bmjopen-2025-101592.reviewer_comments.pdf]

PEER REVIEW HISTORY

BMJ Open publishes all reviews undertaken for accepted manuscripts. Reviewers are asked to complete a checklist review form and are provided with free text boxes to elaborate on their assessment. These free text comments are reproduced below.

ARTICLE DETAILS

Title (Provisional)

Perinatal outcomes and uptake of RSV vaccine during pregnancy in South London: a cross-sectional study

Authors

Razai, Mohammad Sharif S; Kalafat, E.; PRASAD, SMRITI; Lee-Wo, Chelone; Heath, Paul T; Khalil, Asma

---

VERSION 1 - REVIEW

**Reviewer**	**1**
**Name**	**DeSilva, Malini B.**
**Affiliation**	**HealthPartners Institute for Medical Education, Research**
**Date**	**16-Apr-2025**
**COI**	**None**

This manuscript provides an estimate of RSV vaccine uptake among women delivering at a tertiary hospital outside of London along with evaluation of certain vaccine safety outcomes. The inclusion of vaccine safety outcomes seems as it was an after thought as the study is not adequately designed to evaluate these outcomes nor are they thoroughly explained. It would be improved by the following:

- Did RSV vaccination stop after 12/17/24, why did the study not include the entire month of December?

- Does 36 weeks or less include those up to 36 weeks 6 days?

- By including those at 36 weeks or less on 9/1/24, those at later gestational ages had less time to receive the vaccine if they did not get it during the first week or two of availability. Did you account for this immortal time bias when evaluating uptake? It may be helpful to show the distribution of gestational ages at vaccination that occurred stratified by month.

- Is the BMI reported pre-pregnancy BMI? Similar for Obesity, is this defined prior to pregnancy?

- Lines 118 - 121, at what point in pregnancy were alcohol consumption, HDP, and diabetes assessed? It is unclear if this was at any time during pregnancy or prior to vaccination?

- A simple comparison of rates is not adequate for evaluation of vaccine safety outcomes. While the comparisons may not be statistically significant, these unadjusted estimates do show some imbalances between groups and further analysis with robust methods to account for confounding are needed.

- The methods only state that perinatal outcomes were evaluated, but does not explain which outcomes or how these were identified. Please add this to the methods section.

- The conclusion that vaccination was not associated with adverse perinatal outcomes seems to be a step too far from what the results show. The results show some significant differences in rates of perinatal outcomes between group. This could be acknowledged

- An expansion of the limitations section is warranted including the lack of appropriate study design to effectively evaluate vaccine safety outcomes

---

**Reviewer**	**2**
**Name**	**Blauvelt, Christine A.**
**Affiliation**	**University of California San Francisco, Obstetrics, Gynecology and Reproductive Sciences**
**Date**	**19-May-2025**
**COI**	**None**

Summary: This retrospective study evaluates uptake of the prenatal RSVpreF vaccine among pregnant individuals delivering at a single tertiary care hospital in the UK. The manuscript offers valuable early real-world data on vaccine implementation and uptake in the UK setting. A major limitation, however, is the absence of a time-adjusted analytic approach for obstetric outcomes, particularly preterm birth, where differential exposure windows may introduce bias. Additionally, the inclusion of neonatal outcomes related to RSV infection—such as emergency department visits or hospitalizations—would enhance the manuscript's relevance by providing early insights into the real-world effectiveness of prenatal RSV vaccination in preventing neonatal RSV-related morbidity.

Major Comments:

1. Line 127: A key limitation of the current manuscript is the absence of a time-adjusted statistical analysis for preterm birth. Individuals who deliver preterm inherently have less opportunity to receive the RSVpreF vaccine compared to those who deliver at term. As such, a comparison of crude preterm birth rates between vaccinated and unvaccinated groups may be misleading.

2. Line 161: The statement regarding a potential protective effect of RSVpreF vaccination against preterm birth should be interpreted with caution given the limitations of the current analytic approach. Without a time-adjusted model, it is not possible to determine whether the observed association reflects a true effect or is driven by differences in exposure opportunity. The authors should consider removing this interpretation unless further modeling is performed.

3. Line 162: The comparison to prior studies on HDP risk, which employed time-dependent modeling, highlights a missed opportunity for deeper analysis. Since gestational age is a strong determinant of HDP, not using a time-adjusted approach may obscure differences.

4. Can the authors provide more detail regarding the clinical context in which the RSVpreF vaccine was offered? Were vaccines administered in prenatal care clinics, pharmacies, or other outpatient venues? Were there differences in vaccine uptake by type of prenatal care provider (i.e. midwife, obstetrician physician, family medicine physician?) Were there differences in vaccine uptake by prenatal clinic or type of prenatal provider? These data may help identify patterns in vaccine acceptance or access across the study population.

5. The authors could consider expanding the study to include neonatal outcomes such as RSV-related emergency department visits or hospitalizations, comparing infants of vaccinated and unvaccinated individuals. This addition would significantly enhance the clinical impact and novelty of the manuscript.

6. Is nirsevimab (Beyfortus) or palivizumab (Synagis) available in the UK? If so, the authors could consider reporting uptake rates among neonates of unvaccinated mothers.

Minor Comments:

1. The manuscript would benefit from reporting the mean or median gestational age at delivery for each group, as well as the etiology of preterm delivery for those who delivered preterm.

2. Race and ethnicity terms should be capitalized consistently (e.g., “Black,” “White,” “Asian”).

3. Please specify the reference group used in multivariable analyses involving ethnicity.

VERSION 1 - AUTHOR RESPONSE

COMMENT	RESPONSE	EDITS IN MANUSCRIPT – marked copy
**Reviewer 1:**	**Response**	**Edits in manuscript – marked copy**

This manuscript provides an estimate of RSV vaccine uptake among women delivering at a tertiary hospital outside of London along with evaluation of certain vaccine safety outcomes. The inclusion of vaccine safety outcomes seems as it was an after thought as the study is not adequately designed to evaluate these outcomes nor are they thoroughly explained.<br><br>It would be improved by the following:<br><br>- Did RSV vaccination stop after 12/17/24, why did the study not include the entire month of December?<br><br>-Does 36 weeks or less include those up to 36 weeks 6 days?	Thank you for your feedback. We have addressed the points below as suggested and included in the limitations about vaccine safety outcomes.<br><br>The time period for the study was chosen to align with the RSV season and to capture variations in the intake. The RSV vaccination programme is ongoing in line with national guidance. Many vaccination sites operated at a significantly reduced capacity during holiday period 18-31 December.<br><br>It means ≤ 36weeks 0 days	

- By including those at 36 weeks or less on 9/1/24, those at later gestational ages had less time to receive the vaccine if they did not get it during the first week or two of availability. Did you account for this immortal time bias when evaluating uptake? It may be helpful to show the distribution of gestational ages at vaccination that occurred stratified by month.	We have acknowledged the limitation of the study as it does not account for immortal time bias.	Pages 2 and 7
- Is the BMI reported pre-pregnancy BMI? Similar for Obesity, is this defined prior to pregnancy?	We have added this as suggested. BMI has been defined at the first hospital-based antenatal appointment during pregnancy called booking visit in the UK.	Page 4
- Lines 118 - 121, at what point in pregnancy were alcohol consumption, HDP, and diabetes assessed? It is unclear if this was at any time during pregnancy or prior to vaccination?	Alcohol consumption, HDP, and diabetes have been assessed at the booking visit.	Page 4
- A simple comparison of rates is not adequate for evaluation of vaccine safety outcomes. While the comparisons may not be statistically significant, these unadjusted estimates do show some imbalances between groups and further analysis with robust methods to account for confounding are needed.	We have added this in the discussion section as limitations as suggested.	Page 7

- The methods only state that perinatal outcomes were evaluated, but does not explain which outcomes or how these were identified. Please add this to the methods section.	We have added the following in the methods section as suggested: "Perinatal outcomes included preterm birth (<37, <34, and <32 weeks), hypertensive disorders of pregnancy, mode of delivery, intrapartum chorioamnionitis, intrapartum pyrexia, placental abruption, postpartum haemorrhage, maternal admission to a high dependency unit, fetal abnormalities, small-for-gestational age newborn, stillbirth, and neonatal unit admission."	Page 4
- The conclusion that vaccination was not associated with adverse perinatal outcomes seems to be a step too far from what the results show. The results show some significant differences in rates of perinatal outcomes between group. This could be acknowledged.	We have acknowledged in the	Pages 6 and 7

- An expansion of the limitations section is warranted including the lack of appropriate study design to effectively evaluate vaccine safety outcomes	limitations as suggested.<br><br>Thank you. We have expanded the limitations section to address the points you have raised.	Page 7
**Reviewer 2:**		**Edits in manuscript**
Summary: This retrospective study evaluates uptake of the prenatal RSVpreF vaccine among pregnant individuals delivering at a single tertiary care hospital in the UK. The manuscript offers valuable early real-world data on vaccine implementation and uptake in the UK setting. A major limitation, however, is the absence of a time-adjusted analytic approach for obstetric outcomes, particularly preterm birth, where differential exposure windows may introduce bias. Additionally, the inclusion of neonatal outcomes related to RSV infection—such as emergency department visits or hospitalizations—would enhance the manuscript's relevance by providing early insights into the real-world effectiveness of prenatal RSV vaccination in preventing neonatal RSV-related morbidity.<br><br>Major Comments:	Thank you for the feedback. We have revised the manuscript to address the points you have raised as much as possible.	

1. Line 127: A key limitation of the current manuscript is the absence of a time-adjusted statistical analysis for preterm birth. Individuals who deliver preterm inherently have less opportunity to receive the RSVpreF vaccine compared to those who deliver at term. As such, a comparison of crude preterm birth rates between vaccinated and unvaccinated groups may be misleading.	We have acknowledged this in the limitations section.	Pages 2 and 7
2. Line 161: The statement regarding a potential protective effect of RSVpreF vaccination against preterm birth should be interpreted with caution given the limitations of the current analytic approach. Without a time-adjusted model, it is not possible to determine whether the observed association reflects a true effect or is driven by differences in exposure opportunity. The authors should consider removing this interpretation unless further modeling is performed.	We have acknowledged this in the limitations section.	Page 7
3. Line 162: The comparison to prior studies on HDP risk, which employed time-dependent modeling, highlights a missed opportunity for deeper analysis. Since gestational age is a strong determinant of HDP, not using a time-adjusted approach may obscure differences.	We have considered this in the limitations section.	Page 7
4. Can the authors provide more detail regarding the clinical context in which the RSVpreF vaccine was offered? Were vaccines administered in prenatal care clinics, pharmacies, or other		Page 4

outpatient venues? Were there differences in vaccine uptake by type of prenatal care provider (i.e. midwife, obstetrician physician, family medicine physician?) Were there differences in vaccine uptake by prenatal clinic or type of prenatal provider? These data may help identify patterns in vaccine acceptance or access across the study population.	As suggested, we have provided more context for the RSV vaccination. However, since this is a new vaccine, details of uptake between various sites are not yet available.	
5. The authors could consider expanding the study to include neonatal outcomes such as RSV-related emergency department visits or hospitalizations, comparing infants of vaccinated and unvaccinated individuals. This addition would significantly enhance the clinical impact and novelty of the manuscript.	Thank you. We have included this in the limitations as granular data on infant outcomes were not available for analysis.	Page 7
6. Is nirsevimab (Beyfortus) or palivizumab (Synagis) available in the UK? If so, the authors could consider reporting uptake rates among neonates of unvaccinated mothers.	We have included information on nirsevimab (Beyfortus) and palivizumab (Synagis) in the introduction. Nirsevimab is not yet available in the UK.	Page 3

Minor Comments:<br><br>1. The manuscript would benefit from reporting the mean or median gestational age at delivery for each group, as well as the etiology of preterm delivery for those who delivered preterm.	We have reported median gestational age at delivery for each group as follows: "Median gestational age at delivery was similar between RSV and non-RSV vaccinated women: 39.15 weeks (IQR 38.00–40.00) vs 39.12 weeks (IQR 38.00–40.00), respectively (p = 0.056)."<br><br>The antenatal sites do not routinely collect etiology of preterm birth.	Page 5
2. Race and ethnicity terms should be capitalized consistently (e.g., "Black," "White," "Asian").	"Black", "White" and "Asian"	Throughout

	ethnicities/races are capitalised consistently as suggested.	
3. Please specify the reference group used in multivariable analyses involving ethnicity.	We have added that "White" ethnicity was the reference group.	Page 5

VERSION 2 - REVIEW

**Reviewer**	**1**
**Name**	**DeSilva, Malini B.**
**Affiliation**	**HealthPartners Institute for Medical Education, Research**
**Date**	**10-Jun-2025**
**COI**	

This report describes uptake of RSV vaccine during pregnancy in a UK population and attempts to compare perinatal outcomes between vaccinated and unvaccinated women. However, as the authors note, "the study was not prospectively designed or powered to assess vaccine safety, and associations should therefore be interpreted with caution." There are multiple methodological issues with the way the safety assessments were performed and the results are therefore extremely difficult to interpret. I would suggest removing the safety analysis from this paper and only focusing on vaccine uptake. The paper would be improved by providing more information about the approval of RSVpreF,

the rollout at the health system being studied, and further refining the population in whom vaccine uptake is assessed. Is it possible to provide updated data which includes information on a cohort of persons who all delivered and therefore the results are less subject to immortal time bias? If this were possible, I would also suggest modifying the inclusion criteria to reduce immortal time bias by allowing all in the cohort similar exposure risk. Additonally, I have the following comments:

- In the introduction, need to define RSVpreF and specify it is the only RSV vaccine recommended for use in the UK

- Need to describe if the RSV vaccine is recommended seasonally, or at any time of year

- If RSV disease typically peaks in December, why was only half of December included in the evaluation?

-Immortal time bia may also be related to the imbalance in preterm births with no preterm births <34 weeks in the vaccinated group. This should at least be acknowledged.

- If safety outcomes remain in the manuscript, they need to be clearly defined (e.g., what is included in hypertensive disorders of pregnancy and how were they identified, by codes, by vital signs and lab data, by chart review, other; for small for gestational age, what reference standard was used?)

- Given that vaccination uptake increased throughout the time period, were there access issues that impacted uptake?

VERSION 2 - AUTHOR RESPONSE

COMMENT	RESPONSE	EDITS IN MANUSCRIPT – marked copy
**Reviewer comments**		
This report describes uptake of RSV vaccine during pregnancy in a UK population and attempts to compare perinatal outcomes between vaccinated and unvaccinated women. However, as the authors note, "the study was not prospectively designed or powered to assess vaccine safety, and associations should therefore be interpreted with caution." There are	Thank you for your thoughtful and constructive comments. We fully agree that the study was not designed or powered to assess vaccine safety, and we have taken care to reflect this appropriately in the	Pages 1, 4 and 8

multiple methodological issues with the way the safety assessments were performed and the results are therefore extremely difficult to interpret. I would suggest removing the safety analysis from this paper and only focusing on vaccine uptake.	revised manuscript. In response to your suggestion, we have removed the comparison of perinatal outcomes from the stated objectives and have avoided any conclusive statements about safety.<br>We have retained the observational data on perinatal outcomes in the results and conclusions, but presented them in descriptive terms, without making inferential claims. The conclusions now state that observed outcomes did not indicate any apparent adverse patterns among vaccinated women, with a clear emphasis that further research is needed. We hope this addresses your concerns while allowing readers access to all observed data in a transparent and appropriately cautious way	
The paper would be improved by providing more information about the approval of RSVpreF, the rollout at the health system being studied, and further refining the population in whom vaccine uptake is assessed. Is it possible to provide updated data which includes information on a cohort of persons who all delivered and therefore the results are less subject to immortal time bias? If this were possible, I would also suggest modifying the inclusion criteria to reduce immortal time bias by allowing all in the cohort similar exposure risk.<br><br>Additionally, I have the following comments:	Thank you for your helpful comment. As suggested, we have updated the introduction to include additional details about the approval of Abrysvo® (RSVpreF) by the MHRA in November 2023 and its subsequent rollout in the NHS as part of routine antenatal care. We have also added information about the population served by the hospital in the Methods section to clarify the study context.	Pages 3 and 4

- In the introduction, need to define RSVpreF and specify it is the only RSV vaccine recommended for use in the UK	Thank you. As suggested, we have added the following in the introduction: "Abrysvo® (RSVpreF), the bivalent RSV vaccine developed by Pfizer, is the only maternal vaccine licensed in the UK by the Medicines and Healthcare products Regulatory Agency (MHRA) since November 2023 following phase 3 clinical trials."	Page 3
- Need to describe if the RSV vaccine is recommended seasonally, or at any time of year	Thank you. As suggested, we have added the following to the introduction: "RSV vaccination in pregnancy is not seasonal and can be offered year-round to individuals who are at least 28 weeks pregnant."	Page 3
- If RSV disease typically peaks in December, why was only half of December included in the evaluation?	Thank you for your comment. While RSV activity does typically peak in December, routine antenatal services (including booking appointments and walk-in clinics) are severely reduced during the Christmas holiday period. Many women are also less likely to accept vaccination during this time due to increased concern about potential side effects interfering with holiday plans. Although vaccination is offered on inpatient wards during planned or emergency admissions, limited staffing during this period means it is not prioritised. For these	

	reasons, we included births only up to 17 December 2024 to ensure more consistent and representative measurement of vaccine uptake during the active rollout period.	
-Immortal time bia may also be related to the imbalance in preterm births with no preterm births <34 weeks in the vaccinated group. This should at least be acknowledged.	Thank you. As suggested, we have added the following: “Immortal time bias may also explain the absence of preterm births <34 weeks in the vaccinated group.”	Page 8
- If safety outcomes remain in the manuscript, they need to be clearly defined (e.g., what is included in hypertensive disorders of pregnancy and how were they identified, by codes, by vital signs and lab data, by chart review, other; for small for gestational age, what reference standard was used?)	As suggested, we have added the following: “Hypertensive disorders of pregnancy were identified through clinician-recorded diagnoses in the electronic medical record, and small for gestational age was defined as birthweight below the 10th percentile using UK-WHO growth charts.	Page 4
- Given that vaccination uptake increased throughout the time period, were there access issues that impacted uptake?	As suggested, we have added the following to the discussion: “Uptake increased over time as vaccine delivery systems became more established. Early in the rollout, limited staff awareness, a lack of training in vaccine preparation, and changing clinic workflows likely contributed to lower uptake.”	Page 7

VERSION 3 - REVIEW

Reviewer 1
Name DeSilva, Malini B.
Affiliation HealthPartners Institute for Medical Education, Research
Date 08-Jul-2025
COI

The authors have resubmitted the manuscript, but have not included a letter detailing the changes they made. The study is able to complete the objective to evaluate uptake of RSV vaccine and association of vaccine uptake with patient characteristics. However, the addition of perinatal outcomes remains problematic. At a minimum the authors should provide more information about perinatal outcome definitions. For example, how was onset of a hypertensive disorder of pregnancy determined and if a hypertensive disorder of pregnancy was identified prior to vaccination, was that person removed from analysis for that outcome? How was chorioamnionitis identified? What definition was used for intrapartum pyrexi, placental abruption, and postpartum haemorrhage? Were any chart reviews conducted? What is included in the fetal abnormalities outcome? Without this added information, it is difficult to interpret the results. While the authors have included more information about immortal time bias impacting their results, additional consideration about whether it is appropriate to include perinatal outcomes given this bias is warranted. Immortal time bias in this study is related to the timing of follow-up. The chance of RSVpreF vaccination increases the longer a pregnancy lasts. Because the cohort is defined by delivery dates, individuals who reached at least 28 weeks gestation were included, however may have delivered shortly after and would have had limited time to receive the RSVpreF vaccine. Thus, as stated by the authors, immortal time bias may be related to the lack of preterm births in the vaccinated group. Continuing to include perinatal outcomes without providing clear definitions or appropriate methods for evaluating these outcomes is problematic. I also have the following small suggestions:

- Line 72 – should varies be changed to varied?

- Line 88 – is nirsevimab available now in the UK? It would be helpful to explicitly state if it is available or not and if it is available, when it became available

- Line 209 – only one prior study was cited, therefore, would change to "whereas a prior study…"

- Lines 210 – 212 – it is not clear if the authors are referring to their study or the study cited in the previous sentence. Would recommend specifically stating to which study they are referring.

VERSION 3 - AUTHOR RESPONSE

COMMENT	RESPONSE	EDITS IN MANUSCRIPT – marked copy
**Reviewer comments**		
While the authors have modified the manuscript so that it no longer focuses on vaccine safety as a stated objective, they have decided to retain the perinatal outcomes in the manuscript. The study is able to complete the objective to evaluate uptake of RSV vaccine and association of vaccine uptake with patient characteristics. However, keeping perinatal outcomes remains problematic. At a minimum the authors should provide more information about perinatal outcome definitions. For example, how was onset of a hypertensive disorder of pregnancy determined and if a hypertensive disorder of pregnancy was identified prior to vaccination, was that person removed from analysis for that outcome? How was	Thank you for your detailed and constructive comments. We have revised the manuscript accordingly and added further information regarding the perinatal outcome definitions, including references where applicable:<br>• Hypertensive disorders of pregnancy were identified through clinician-recorded diagnoses in the electronic medical record, based on the 2021 International Society for the Study of Hypertension in Pregnancy (ISSHP) guidelines.<br>• Small for gestational age was classified as a birth weight below the 10th percentile for gestational age, using the UK-WHO growth charts.<br>• Intrapartum chorioamnionitis was characterised by a combination of clinical indicators, including maternal tachycardia, a maternal temperature of ≥38.0°C on a single reading or ≥37.5°C on two consecutive occasions at least one	Pages 4-5 (Lines 117-134)

chorioamnionitis identified? What definition was used for intrapartum pyrexi, placental abruption, and postpartum haemorrhage? Were any chart reviews conducted? What is included in the fetal abnormalities outcome? Without this added information, it is difficult to interpret the perinatal results.	hour apart, elevated inflammatory markers, and fetal tachycardia.<br>• Postpartum haemorrhage was defined as blood loss of 500 mL or more from the genital tract following childbirth.<br>• Placental abruption was diagnosed clinically, in line with the Royal College of Obstetricians and Gynaecologists (RCOG) guidance on the management of antepartum haemorrhage.<br>• Fetal abnormalities included conditions screened for under the NHS Fetal Anomaly Screening Programme (FASP), which assesses for 14 anomalies, including structural abnormalities (e.g. open spina bifida, congenital diaphragmatic hernia, major cardiac defects) and chromosomal abnormalities (trisomy 21, 18, and 13).[18]<br>• Stillbirth was defined as fetal death at ≥24 weeks' gestation.<br><br>All outcomes were derived from routinely recorded structured clinical data within the electronic health record.	
While the authors have included more information about immortal time bias impacting their results, additional consideration about whether it is appropriate to include perinatal outcomes given this bias is warranted. Immortal time bias in this study is related to the timing of follow-up. The chance of RSVpreF vaccination increases the longer a pregnancy lasts. Because the cohort is defined by delivery dates, individuals who reached at least 28 weeks gestation were included, however may have delivered shortly after and would have had limited time to receive the	We thank the reviewer for this important observation. We agree that immortal time bias, arising from the cohort definition based on delivery dates and variation in gestational age at vaccination opportunity, may influence the interpretation of perinatal outcomes. As stated in the limitations section, this bias may explain the absence of preterm births <34 weeks in the vaccinated group. We have now strengthened this limitation by explicitly noting that perinatal outcome comparisons, particularly for gestational age sensitive outcomes, must be interpreted with caution. The following is added: "Immortal time bias may also explain the absence of preterm births <34 weeks in the vaccinated group. Given this, comparisons of perinatal outcomes, particularly those sensitive to gestational age, should be interpreted	Page 8 (Lines 255 – 258)

RSVpreF vaccine. Thus, as stated by the authors, immortal time bias may be related to the lack of preterm births in the vaccinated group. Continuing to include perinatal outcomes without providing clear definitions or appropriate methods for evaluating these outcomes is problematic.	with caution, as this bias may have influenced the observed differences between groups.”	
I also have the following small suggestions:		
- Line 72 – should varies be changed to varied?	Thank you for the suggestion. We have reviewed the sentence and believe that "varies" is appropriate in this context, as the implementation of RSV vaccination in the EU region is ongoing and subject to differences across countries. Therefore, the present tense reflects the current and changing nature of the process.	
- Line 88 – is nirsevimab available now in the UK? It would be helpful to explicitly state if it is available or not and if it is available, when it became available	As suggested, we have added: “Nirsevimab is not currently available through a national immunisation programme in the UK.”	Page 3 (Lines 89 – 90)
- Line 209 – only one prior study was cited, therefore, would change to “whereas a prior study…”	As suggested, we have corrected this to: “whereas a prior study…”	Page 7 line 223
- Lines 210 – 212 – it is not clear if the authors are referring to their study or the study cited in the	Thank you. We have clarified this to mean our study and strengthened the limitations.	Page 7 (Lines 222 – 226)

previous sentence. Would recommend specifically stating to which study they are referring.		

VERSION 4 - REVIEW

Reviewer 1

Name DeSilva, Malini B.

Affiliation HealthPartners Institute for Medical Education, Research

Date 31-Jul-2025

COI

This is a revised manuscript evaluating uptake of RSV vaccine during pregnancy in the UK during the first few months of use. As additional time has passed, would it be possible to expand the time period of the study? While the authors have addressed some of the previous comments, the lack of complete details about the perinatal outcomes evaluated remains problematic. The hypertensive disorders of pregnancy are noted to be diagnosed according to the 2021 International Society for the Study of Hypertension in Pregnancy guidelines, however there is no mention of chart review to determine if this is accurate. Similarly, intrapartum chorioamnionitis is noted to be characterized by various clinical factors, however the data source is not explained to understand how these clinical factors are abstracted. Finally, hypertensive disorders of pregnancy are listed as clinical characteristics in Table 1 and Table 2 and are also listed as outcomes. The percentage of those with a hypertensive disorder of pregnancy listed in Table 1 is different from what is listed in the results. It is unclear if hypertensive disorders of pregnancy are assessed prior to vaccination or after. More needs to be added to the methods so that the study could be replicated by an outsider. Given the substantial limitations related to the perinatal outcomes, it may be best to remove them from the manuscript. Additionally, as more time has now elapsed, expanding the timeframe to understand vaccine uptake across a larger time period would be helpful to understand how uptake may have changed over time.

VERSION 4 - AUTHOR RESPONSE

COMMENT	RESPONSE	EDITS IN MANUSCRIPT – marked copy
**Reviewer comments**		
This is a revised manuscript evaluating uptake of RSV vaccine during pregnancy in the UK during the first few months of use. As additional time has passed, would it be possible to expand the time period of the study?	Thank you very much for this thoughtful suggestion. We agree that a longer observation period would be valuable; however, our analysis is based on data available. We hope to explore trends over an extended timeframe in future.	
While the authors have addressed some of the previous comments, the lack of complete details about the perinatal outcomes evaluated remains problematic. The hypertensive disorders of pregnancy are noted to be diagnosed according to the 2021 International Society for the Study of Hypertension in Pregnancy guidelines, however there is no mention of chart review to determine if this is accurate.	Thank you for this constructive and helpful feedback. We have added chart review in the method section as suggested. We apologise that it was missed in our previous revision. We have added: "To ensure accuracy, a chart review was conducted to confirm that the documented diagnoses met the criteria outlined in the guidelines."	Page 4 lines 119-121
Similarly, intrapartum chorioamnionitis is noted to be characterized by various clinical factors, however the data source is not explained to understand how these clinical factors are abstracted.	Thank you for your comment. We revised this section as suggested to improve clarity. Intrapartum chorioamnionitis was identified based on a clinician-assigned diagnosis coded in the electronic medical records. The diagnosis was made using clinical observations such as maternal temperature, tachycardia, raised inflammatory markers, and fetal	Pages 4-5 lines 127-130

	tachycardia, in accordance with national guidance, which we have described in the manuscript.	
Finally, hypertensive disorders of pregnancy are listed as clinical characteristics in Table 1 and Table 2 and are also listed as outcomes. The percentage of those with a hypertensive disorder of pregnancy listed in Table 1 is different from what is listed in the results. It is unclear if hypertensive disorders of pregnancy are assessed prior to vaccination or after. More needs to be added to the methods so that the study could be replicated by an outsider.	We thank the reviewer for this comment. In our cross-sectional study, all variables — including hypertensive disorders of pregnancy (HDP) — were obtained for the entire pregnancy after delivery. HDP appears in Table 1 to show its distribution between vaccinated and unvaccinated women, and in Table 2 to explore whether HDP was associated with vaccine uptake. We did not evaluate HDP in relation to the timing of vaccination, and no temporal or causal inference can be made from these data. We have added clarifying text to the Methods to make this explicit. The small difference in percentages between the table and text was due to rounding and has now been corrected so that the figures match.	Page 4 lines 121-125
Given the substantial limitations related to the perinatal outcomes, it may be best to remove them from the manuscript.	Thank you for your comment. As is the usual practice, we have not completely excluded data due to limitations. Instead, we have clearly and transparently outlined the relevant caveats in the manuscript, allowing readers to interpret the findings with appropriate caution.	Pages 7-8
Additionally, as more time has now elapsed, expanding the timeframe to understand vaccine uptake across a larger time period would be helpful to understand how uptake may have changed over time.	Thank you for this helpful suggestion. We agree that examining uptake over a longer period would provide useful insights into temporal trends. While	

	our current analysis reflects the data available, we plan to explore longer-term patterns in future work.	

VERSION 5 - REVIEW

Reviewer 1

Name DeSilva, Malini B.

Affiliation HealthPartners Institute for Medical Education, Research

Date 12-Aug-2025

COI

The authors have made substantial changes to previous reviews. In the abstract I would recommend removing, "Observed perinatal outcomes did not indicate any apparent adverse patterns among vaccinated women," as the study was not designed to specifically evaluate perinatal outcomes. While this limitation is addressed in the main paper, there is no mention of the limitations of evaluating these outcomes in the abstract.

VERSION 5 - AUTHOR RESPONSE

response to the points raised.

COMMENT	RESPONSE	EDITS IN MANUSCRIPT – marked copy

**Reviewer comments**		
The authors have made substantial changes to previous reviews. In the abstract I would recommend removing, "Observed perinatal outcomes did not indicate any apparent adverse patterns among vaccinated women," as the study was not designed to specifically evaluate perinatal outcomes. While this limitation is addressed in the main paper, there is no mention of the limitations of evaluating these outcomes in the abstract.	Thank you for your helpful feedback and suggestions, which have greatly improved the manuscript. We have removed the sentence from the abstract as requested.	Abstract lines 27-29
